# L-2 hydroxyglutaric aciduria: report of a Mexican-Mayan patient with the mutation c.569C>T and response to vitamin supplements and levocarnitine

**DOI:** 10.5334/tohm.854

**Published:** 2024-03-07

**Authors:** Roberto Leal-Ortega, Luis Enrique Parra-Medina, Lizbeth Josefina González-Herrera

**Affiliations:** 1 Department of Neurology, Hospital Regional de Alta Especialidad de la Peninsula de Yucatán. Mérida, Mexico; 2 Department of Neurology, Instituto Nacional de Neurología y Neurocirugía “Manuel Velasco Suárez”. Mexico City, Mexico; 3 DIMYGEN Laboratorio S.C.P, Mérida, Yucatán, México; 4 Laboratory of Genetics. Centro de Investigaciones Regionales “Dr. Hideyo Noguchi”, Universidad Autónoma de Yucatán. Mérida, Mexico

**Keywords:** L-2 hydroxyglutaric aciduria, L2HGDH, cerebellar ataxia, tremor, T2-hyperintensities, riboflavin, L-carnitine

## Abstract

**Background:**

L-2-hydroxyglutaric aciduria (L2HGA) is a rare inherited autosomal recessive neurometabolic disorder caused by pathogenic variants in the *L2HGDH* gene which encodes mitochondrial 2-hydroxyglutarate dehydrogenase. Here, we report a case of L2HGA in a Mexican-Mayan patient with a homozygous mutation at *L2HGDH* gene and clinical response to vitamin supplements and levocarnitine.

**Case report:**

A 17-year-old, right-handed female patient with long-term history of seizures, developmental delay and ataxia was referred to a movement disorders specialist for the evaluation of tremor. Her brain MRI showed typical findings of L2HGA. The diagnosis was corroborated with elevated levels of 2-hydroxyglutaric acid in urine and genetic test which revealed a homozygous genetic known variant c.569C>T in exon 5 of *L2HGDH* gene. She was treated with levocarnitine and vitamin supplements, showing improvement in tremor and gait.

**Discussion:**

To our knowledge this is the first report of a Mexican patient with L2HGA. This case adds information about a rare condition in a different ethnic group and supports the findings of other authors which encountered symptomatic improvement with the use of flavin adenine dinucleotide (and its precursor riboflavin), and levocarnitine.

**Highlights:**

We report the first case of Mexican-Mayan patient with L2HGA showing a missense homozygous mutation in *L2HGDH* gene, and improvement of symptoms with vitamin supplements and levocarnitine.

## Background

L-2-hydroxyglutaric aciduria (L2HGA) is a rare inherited autosomal recessive neurometabolic disorder caused by pathogenic variants in the *L2HGDH* gene (14q.22.1) which encodes mitochondrial 2-hydroxyglutarate dehydrogenase [1]. To date, very few cases (around 150) have been reported worldwide of this condition [2]. L2HGA is a slowly progressive illness starting in childhood with symptoms including cognitive delay, seizures, macrocephaly, and movement disorders such as tremor dystonia, and cerebellar ataxia [3]. Often, patients remain undiagnosed until adolescence or adulthood. Brain MRI of patients with L2HGA can provide important clues to diagnosis. T2 hyperintensities with centripetal extension predominantly in the anterior white matter involving the subcortical U-fibers and sparing the brainstem and corpus callosum are typical; cerebellar atrophy and hyperintensities in lentiform nucleus, caudate and dentate nucleus are also described [4]. Besides the MRI findings, elevation of L-2-hydroxyglutaric acid in urine, plasma and cerebrospinal fluid, and mutational analysis of the *L2HGDH* gene confirm the diagnosis [1,2,3]. No specific treatment exists for this condition and supportive measures include treatment of seizures, cognitive and physical rehabilitation. Some case reports have described improvement with riboflavin and L-carnitine supplementation [1,5]. Here we report the first case of L2HGA in a 17-year-old Mexican-Mayan female presenting with cerebellar syndrome, seizures and cognitive impairment. She was diagnosed with L2HGA based on MRI findings, high levels of L-2- hydroxyglutaric acid in urine, and mutation of *L2HGDH* gene. Also, we documented improvement of movement disorders with use of L-carnitine and vitamins/minerals supplementation.

## Case report

A 17-year-old, right-handed female patient was referred to a movement disorders specialist to evaluate upper extremities tremor. Her parents were healthy, apparently non– consanguineous, originated from southeast Mexico in the Mayan area of Yucatan Peninsula. She was born after an uneventful pregnancy and normal childbirth. At 13 months, she started with recurrent non-febrile generalized tonic-clonic seizures. Since then, she experienced psychomotor developmental delay, exhibiting the ability to walk at the age of 4 years and incomplete language development at 5 years. Schooling was delayed and received assistance from special schools and ongoing physical and cognitive therapy. At that time, she had a non-specific diagnosis of generalized epilepsy and global development delay. During next ten years she experienced gradually neuropsychiatric symptoms including anxiety, aggressive behaviors to family members, self-injury, and irregular sleep pattern. At the age of 15, she developed upper extremity tremor and gait instability, and she was referred to a movement disorders specialist. She was on chronic treatment with levetiracetam 2 g/day and risperidone 2 mg/day.

**Figure 1:**
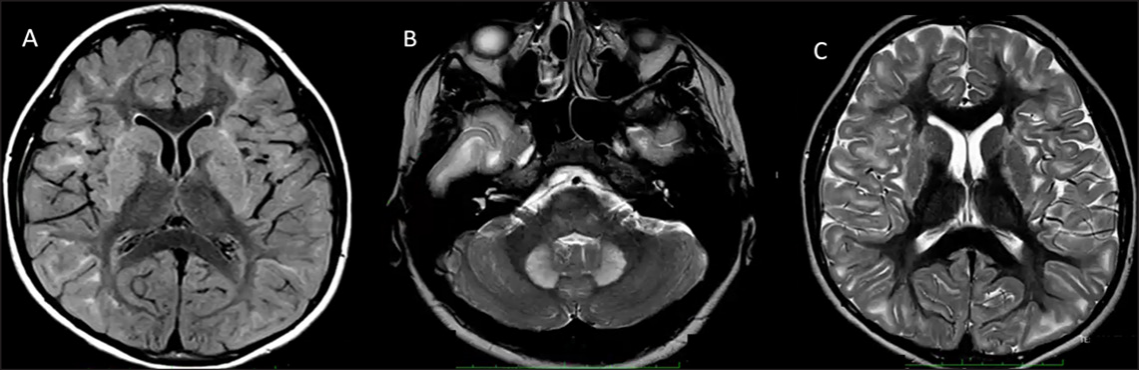
Axial brain MRI sequences. **(A)** FLAIR sequence shows bilateral subcortical white matter hyperintensities in frontal – parietal and temporal lobes. **(B)** T2WI with bilateral hyperintensities of dentate nuclei, and **(C)** T2WI showing bilateral hyperintensities in basal ganglia. Note in **(A)** and **(C)** the sparing of both thalami.

Her general examination was normal including head circumference. The neurological examination revealed cognitive impairment, slurred speech, hypometric horizontal saccades spasticity in lower extremities, strength grade 4 of 5 in the Medical Research Council scale in the major muscles of four extremities; hyperreflexia was present in all extremities, with bilateral extensor plantar response; rest and action tremor, dysdiadochokinesia, and dysmetria on the finger-nose maneuver were present in both upper extremities; her walking was unstable, slow, with slight dragging of the legs, and inability for tandem gait (Video 1). Complete blood count, biochemistry profiles, lactate, thyroid function, and blood ceruloplasmin tests were normal. Brain MRI revealed in T2W, T2-FLAIR and DWI bilateral hyperintensities in the subcortical white matter in fronto-parieto-temporal lobes, and symmetrical hyperintensities in the dentate nuclei and basal ganglia without involvement of thalamus and brainstem (Figure 1). Because of the high suspicion of L2HGA, a urine screening examination was performed using the total ion chromatogram method of urinary metabolites by gas chromatography/mass spectrometry (GC/MS), which revealed elevated excretion of 2-hydroxyglutarate. Genetic test was conducted on peripheral blood using isolated genomic DNA. Sanger sequencing of the *L2HGDH* gene (OMIM 609582, 14q21.3) revealed a homozygous variant c.569C>T in exon 5 of *L2HGDH* gene. A final diagnosis of L2HGA was made and we started supplementation with multivitamins and levocarnitine 3 g/day showing improvement in upper extremities tremor and gait (Video 2). She lost follow up almost two years because of SARS-CoV2 pandemic and stopped the treatment, showing a worsening of motor symptoms. After reinitiation of levocarnitine and multivitamins, she improved again.

**Video 1:** **L Hydroxyglutaric aciduria segment**. Patient manifesting rest, postural and intentional tremor in both upper extremities, dysarthria, dysdiadochokinesia, and unstable gait with dragging of the legs.

**Video 2:** **L2 hydroxyglutaric aciduria segment 2**. Improvement of upper extremities tremor and gait with the use of levocarnitine and vitamin supplements.

## Discussion

Initially described in 1980 by Duran et al in a 5-year-old Moroccan boy with psychomotor delay, dystrophy and large amounts of 2- hydroxyglutaric acid in the urine [6], L2HGA is classified as an organic cerebral aciduria caused by L-2-hydroxyglutarate dehydrogenase enzyme deficiency which is involved in the oxidation of L-2-hydoxyglutarate (L-2-HG) to alpha 2- ketoglutarate. The accumulation of L-2-HG has toxic effects to the brain due to free radical formation and increases of glutamate uptake in synaptosomes and synaptic vesicles, leading to a progressive leukoencephalopathy [2]. Clinical course is usually chronic and gradually progressive with ataxia, spasticity, dystonia, seizures, macrocephaly, and intellectual disability [1,2,3,4,5]. Our case represents a new report of L2HGA in a Mexican patient from the Mayan area of Yucatan. To our knowledge, this is the first report of a Mexican patient and one of the few reports of L2HGA in Latin America. This supports that L2HGA is a rare but pan-ethnic disease, although must of cases have been reported in Turkey. Our patient showed classical MRI findings which was a clue to conduct the next diagnostic studies. Typically, abnormalities of predominantly subcortical cerebral white matter in combination with abnormalities of the dentate nucleus, globus pallidus, putamen, and caudate nucleus has been described in L2HGA [1,2,3,4,5,7] In a large series of 56 patients, the appearance of the MRI abnormalities was characterized by more global and confluent abnormalities of the cerebral white matter, caudate nucleus, and putamen, and by atrophy of the cerebral white matter and cerebellar hemispheres [8]. The differential diagnosis of L2HGA includes D-2 hydroxyglutaric aciduria (D2HGA) and combined L2HGA and D2HG (D, L-2-HGA). The clinical phenotype of D2HGA includes epilepsy, psychomotor delay, cardiomyopathy, facial dysmorphia, dystonia and chorea-athetosis [9]; brain MRI shows enlargement of the lateral ventricles, subdural effusions, subependymal seudocysts, delayed myelinization, and callosal agenesis [1,9]. These radiological signs were not found in our case. D, L-2-HGA is characterized by elevated levels of both D-2-HG and L-2-HG in body fluids; the clinical manifestations include severe neonatal epileptic encephalopathy, absence of developmental progress, and often early death [10]. Impaired function of the mitochondrial citrate carrier due to pathogenic mutations within the SLC25A1gene has been identified as the underlying molecular cause of the disease [11]. The clinical course described in D, L-2-HGA was not compatible with our patient.

Patients with L2HGA have a predisposition to cerebral neoplasms. This may be related to the pathologic accumulation of L2HGA because high amounts of 2-HG have been found in brain neoplasms like astrocytoma, medulloblastoma, ependymoma, oligodendroglioma, and others [12]. Our patient was negative for tumors.

*L2HGA* gene (14q22.1) encodes a putative mitochondrial protein with homology to flavin adenine dinucleotide (FAD) dependent oxidoreductases. So far, the human gene mutation database has reported 52 missense or nonsense mutations implicated in the reduced action of the L2HGDH enzyme [13,14,15]. The genetic variant detected in our patient is a missense mutation leading to a substitution of a threonine by a isoleucine in position 190 of the encoded protein (p.Thr190Ile) lying in the highly conserved FAD-binding domain, disturbing the enzymatic function. Pathogenicity of this genetic variant was predicted to be deleterious, damaging or disease causing by five bioinformatic prediction tools such as *PROVEAN, MutationAssessor, FATHMM-MKL, SIFT*, PolyPhen-2. Clinical variant classification confers it a variant with uncertain significance (VUS). This variant c.569C>T has been only once reported in an Iranian patient showing intellectual disability and epilepsy [16]. Previous studies with extensive families from different populations and ethnicities have not recorded our mutation [7,17]. According to http://www.LOVD.nl/L2HGDH missense mutations are the most frequent changes, in about 32%. The severity of damage to L2HGDH varies with the type of mutation. The type of the amino acid replacement as well as the sum of the bonds and it forces affect the binding and dissociation of the L-2 hydroxyglutaric acid molecule with the L2HGDH enzyme to alter its action [15].

Treatment of L2HGA is symptomatic for seizures, motor, cognitive and behavioral symptoms. Supplementation with FAD and its precursor riboflavin has been reported successful for symptomatic treatment, improving functionality in some cases [2,18,19]. The rationale for its use is the theoretical increase of residual activity of L2HGDH by FAD and riboflavin [20]. Based on these experience, we decided to treat the patient with a daily dose of vitamin supplements (riboflavin content of 3.400 mg) and levocarnitine (3 g) for a period of a year, showing improvement in gait and hands tremor.

This new case of L2HGA in a different ethnic group adds information about a rare condition, being important of having a high index of suspicion in patients with non-defined diagnostic of seizures, cerebellar ataxia, cognitive delay, and typical MRI findings. For symptomatic treatment, the use of riboflavin and levocarnitine should be offered to patients.
